# Sugarcane Pokkah Boeng Disease: Insights and Future Directions for Effective Management

**DOI:** 10.3390/life14121533

**Published:** 2024-11-22

**Authors:** Rajendran Poorniammal, Jerald Jernisha, Somasundaram Prabhu, Laurent Dufossé

**Affiliations:** 1 Department of Agricultural Microbiology, Tamil Nadu Agricultural University, Coimbatore 641 003, Tamil Nadu, India; jernishachristi@gmail.com; 2 Department of Plant Protection, Horticultural College and Research Institute, Tamil Nadu Agricultural University, Periyakulam 625 604, Tamil Nadu, India; prabhu.s@tnau.ac.in; 3 CHEMBIOPRO Lab, Chimie et Biotechnologie des Produits Naturels, ESIROI Agroalimentaire, Université of Réunion Island, 97400 Saint-Denis, France

**Keywords:** *Fusarium* complex, multitrophic interaction, ecology, symptoms, IDM

## Abstract

Pokkah Boeng disease has been observed in nearly all countries where sugarcane is commercially cultivated. The disease was considered a minor concern in earlier times, but due to climate change, it has now become a major issue. It is caused by fungi, specifically the *Fusarium* fungal complex. *Fusarium fujikuroi*, *F. sacchari*, *F. oxysporum*, *F. verticillioides*, *F. proliferatum*, and *F. subglutinans* are the major species causing the disease in sugarcane. The disease spreads rapidly, and unpredictable environmental conditions, along with the overlap of crop stages with biotic factors, contributed to its increased severity and varied symptom patterns. This disease is primarily airborne, spreading through air currents. Secondary transmission occurs via infected setts, irrigation water, splashed rain, and soil. It typically emerges during hot and humid conditions, particularly when the sugarcane is experiencing rapid growth. The most effective way to control Pokkah Boeng is by cultivating resistant varieties and removing canes exhibiting ‘top rot’ or ‘knife cut’ symptoms. Apply 0.1% carbendazim, 0.2% copper oxychloride, or 0.3% mancozeb for two to three sprayings at 15-day intervals. Using biological methods to control plant pathogens presents a promising alternative to the heavy reliance on chemical fungicides in modern agriculture, which can lead to environmental pollution and the development of resistant strains.

## 1. Introduction

Sugarcane is one of India’s most significant cash crops, offering relatively low risk to farmers and providing a dependable return, even under challenging conditions. It serves as the raw material for the country’s second-largest agro-based industry, next only to textiles. The sugar industry plays a vital role in creating substantial employment opportunities in rural areas, both directly and through related sectors. Sugarcane and its derivatives contribute approximately 1.1% to India’s GDP. Over the past two decades, the share of sugarcane in the agricultural GDP has consistently risen. Additionally, the sugarcane and sugar industries are significant sources of employment and livelihood in India. More than 50 million farmers are involved in sugarcane cultivation and processing. Achieving maximum yields from sugarcane crops enhances the economic conditions of these farmers [1].

**Figure 1:**
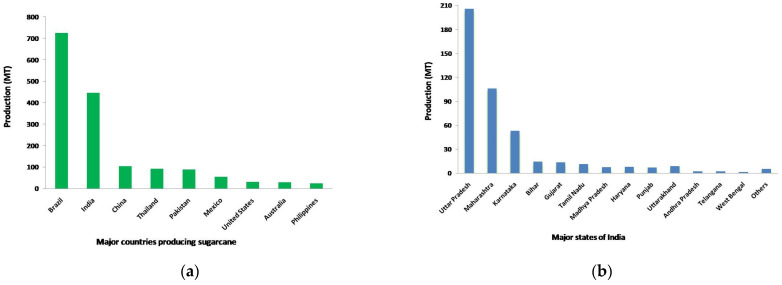
Sugarcane production (MT million tons) from 2019 to 2024 (March) (FAOSTAT, 2024). (**a**) Worldwide sugarcane production; (**b**) Indian state wise sugarcane production.

Sugarcane is an important crop grown in tropical and subtropical regions, covering more than 27 million hectares across 121 countries globally. Brazil, the world’s leading sugarcane producer, and India, the second largest, are projected to contribute approximately 25% and 19% of global sugar production by 2023. The global sugarcane production in India reached as much as 280.79 lakh tons (280,790 million tons) as of March 2024 [2] (Figure 1a).

Approximately 51% of India’s sugarcane production comes from tropical regions such as Maharashtra, Karnataka, Tamil Nadu, and Andhra Pradesh. The remaining 49% is produced in sub-tropical regions, including Uttar Pradesh, Bihar, Uttarakhand, Punjab, and Haryana, even though these sub-tropical areas account for 55% of the total land under sugarcane cultivation [3] (Figure 1b).

Environmental stress can significantly hinder plant growth and yield, especially in sugarcane. Gaining insight into the biochemical and physiological responses of plants to environmental conditions, along with understanding stress mechanisms and developing crop tolerance strategies, is essential to reducing these negative impacts [4]. Sugarcane farming faces numerous challenges from both biotic and abiotic stresses, such as weeds, diseases, insect pests, drought, salinity, cold temperatures, heavy metals, and poor soil fertility.

Abiotic factors such as heat stress can greatly limit plant productivity, reducing the yield of various agricultural species by 2.5% to 10% [5]. Elevated temperatures can adversely affect photosynthesis, respiration, water balance, and membrane stability in leaves, ultimately leading to lower crop yields [6]. Environmental stressors can lead to the denaturation of proteins or enzymes, damage to membranes, increased level s of reactive oxygen species (ROS), cellular disruption, and DNA damage. These factors collectively reduce crop yields and hinder the growth of sugarcane. Heat stress, in particular, can result in drought conditions and increased susceptibility to disease infections [7].

Sugarcane diseases pose significant challenges to crop production globally, with no country protected from the damaging effects of plant pathogens and pests. Over 125 diseases affecting sugarcane, caused by fungi, bacteria, viruses, phytoplasma, and nematodes, have been documented worldwide [8].Pests and diseases, involving around 100 fungi, 10 bacteria, 10 viruses, and 50 nematodes, are significant factors in reducing global crop yields [9]. In sugarcane, diseases such as rust (20% yield loss), smut (75%), ratoon stunting disease (RSD) (40%), and mosaic virus (40%) contribute to a 37% decline in global agricultural production, with an additional 13% of losses attributed to insect damage [10].

Fungal pathogens pose significant challenges among biotic agents, with over 100 fungi known to cause diseases in sugarcane. The most notable diseases include whip smut, red rot, leaf blast, sugarcane mosaic virus, pineapple disease, ratoon stunting disease, leaf scald, mottled stripe, Pokkah Boeng, and wilt [11].

Globally, over 200 diseases have been identified in sugarcane [9], with around 55 of these being economically significant in India [12]. Among the major diseases in India, Pokkah Boeng (PB), a *Fusarium* disease, has been reported in all sugarcane-growing regions and was initially considered minor. The fungus *Fusarium verticillioides* (*Fusarium moniliforme*) (Sacc.) Nirenberg, (1976) is Ascomycetefungi of the order Hypocreales, family Nectriaceae. Reinking (1918) first reported it in the Philippines, infecting corn ears and causing root, stalk, and ear rots (Cumagun, 2008). It infects young leaves, causing malformations and twisted tops during the monsoon season. Typically, affected varieties recover after the monsoon [13]. However, in recent years, PB has become more severe in various states, causing significant damage to cane cultivation [14,15].

The disease led to the removal of several commercial crop varieties from cultivation in regions where it is endemic. Its severity was primarily observed in the east coastal regions, southern Gujarat, and the subtropical plains. In contrast, upland areas in Tamil Nadu, Karnataka, Maharashtra, and Andhra Pradesh experienced little to no severe outbreaks, with only sporadic instances reported [15,16].

## 2. Epidemiology, Ecology, and Dissemination of Pokkah Boeng Pathogens

### 2.1. History

Pokkah Boeng in sugarcane is caused by the fungus *F. moniliforme*, first described by Sheldon. The perfect stage of this pathogen is known as *Gibberella fujikuroi* (Sawada1919). Numerous researchers, the pioneers in identifying the Pokkah Boeng disease in sugarcane, confirmed *Fusarium* as the causal agent of Pokkah Boeng in sugarcane across Asia, establishing it as a significant pathogen in the region. It seems that fungi with slower growth rates, which are less adept at outcompeting faster-growing fungi by occupying specific niches, tend to exhibit a higher degree of antagonism towards their fungal competitors [17]. Pokkah Boeng of sugarcane was first reported in India between July and November of 1983–1984 in Maharashtra, affecting two cane varieties: Co7219 and CoC671. Over time, Pokkah Boeng emerged as one of the major sugarcane diseases in India [18].

*Fusarium* is a highly destructive phytopathogenic fungus classified under the division Ascomycota, class Sordariomycetes, order Hypocreales, and family Nectriaceae. This genus comprises numerous species known to be plant pathogens. The genus *Fusarium* includes 16 recognized sections: *Eupionnotes*, *Macroconia*, *Spirarioides*, *Submicrocera*, *Pseudomicrocera*, *Arachnites*, *Sporotrichiella*, *Roseum*, *Arthrosporiella*, *Gibbosum*, *Discolor*, *Lateritium*, *Liseola*, *Elegans*, *Martiella*, and *Ventricosum*. Many *Fusarium* species are more commonly found in fertile cultivated and rangeland soils rather than forest soils. These species are responsible for various diseases affecting many economically significant cereals and crops [19].

*Fusarium* species are widespread and can persist in soil for extended periods. These fungi typically become problematic following plant stress. It is well established that *Fusarium* causes two distinct diseases: one affecting the stalk and the other targeting the leaves or spindle. The species *Fusarium sacchari*(E.J. Butler and Hafiz Khan and W. Gams 1971) and *F. verticillioides* are specifically associated with these respective diseases [20].

**Figure 2:**
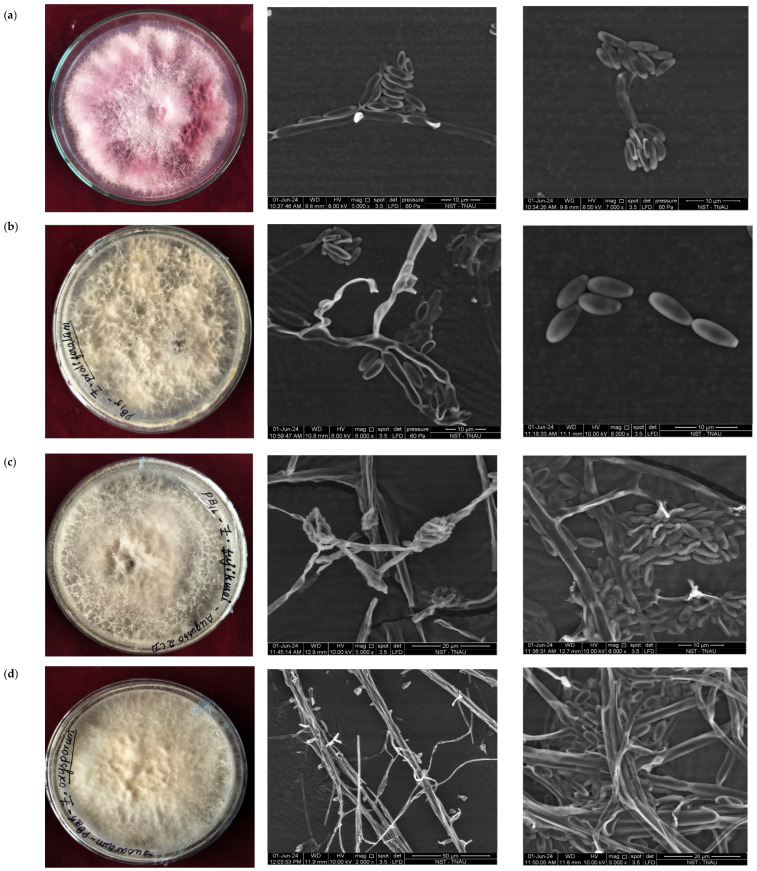
Morphological and conidial characteristics of *Fusarium* sp.(**a**) *F. fujikuroi* strain CSV1 (**b**) *F. proliferatum*, (**c**) *F. fujikuroi* strain Augusto 2, and (**d**) *F. oxysporum*.

*Fusarium verticillioides* (Sacc.) Nirenberg, 1976 is the most frequently reported fungal species infecting sugarcane. This species, previously known as *F.moniliforme*, is capable of producing the chemical fusaric acid. Among the *Fusarium* species, *F. verticillioides* is particularly prominent. *Fusarium proliferatum* (Matsush.) Nirenberg ex Gerlach and Nirenberg, 1982, is another common pathogen that infects a variety of crop plants across different climatic zones. *F. sacchari*, *F. verticillioides*, *F. proliferatum*, and *Fusarium subglutinans* (Wollenw and Reinking) P.E. Nelson, Toussoun, and Marasas 1983 have all been isolated from sugarcane. *F. sacchari* thrives on decaying plant material and produces numerous conidia, which are dispersed by wind and rain [21]. The disease-causing *Fusarium* and its microscopic view shown in Table 1 and Morphology and conidial character of *Fusarium* Pokkah Boeng are shown in Figure 2.

### 2.2. Symptoms and Host Range

**Figure 3:**
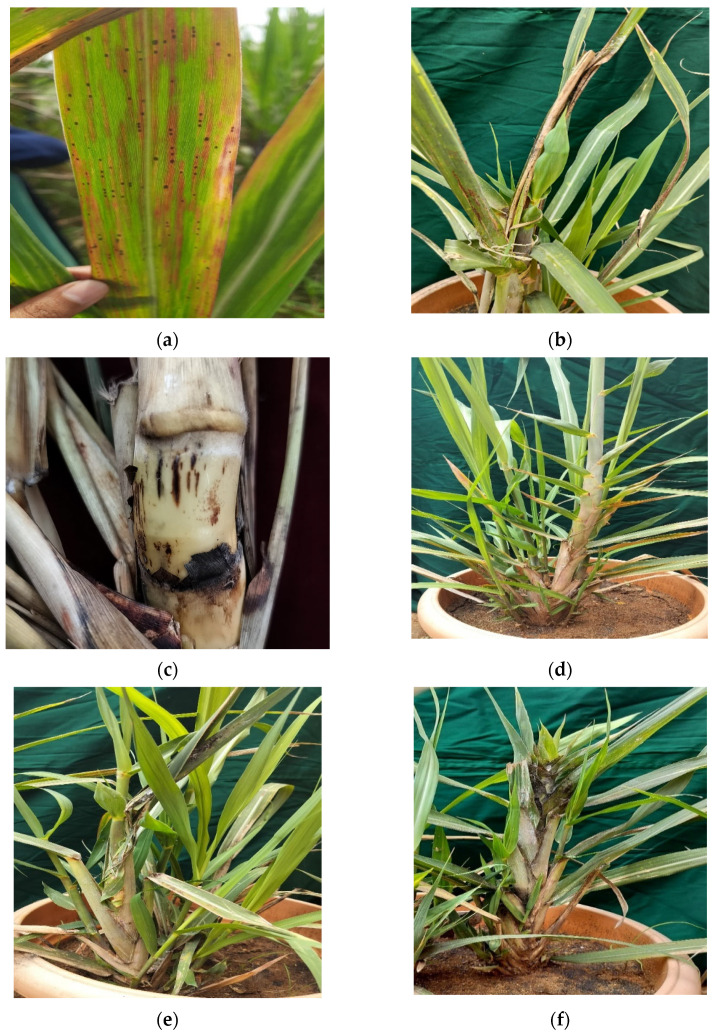
Pokahh Boeng symptoms on sugarcane (**a**) dhlorotic phase, (**b**) top rot phase, (**c**) knife cut phase, (**d**) ladder-like lesions on shoot, (**e**) twisted leafs, and (**f**) malformed shoot.

Pokkah Boeng in sugarcane is common during monsoon/post monsoon months in the field in different parts of the country. It is highly influenced by the favorable climatic conditions viz., high humidity, and temperature [22]. The symptoms of this disease start to develop in 3–5 month-old canes during periods in which rainfall favors the infection of the pathogen [23] (Figure 3).

#### 2.2.1. Chlorotic Stage

The initial sign of Pokkah Boeng appears as a yellowing (chlorosis) near the base of young leaves, sometimes extending to other parts of the leaf blade. This stage often includes noticeable wrinkling, twisting, and shortening of the leaves, along with their abnormal shape or distortion. The base of the affected leaves tends to be narrower compared to healthy leaves [24].

#### 2.2.2. Acute or Top Rot Stage

This is the most severe phase of Pokkah Boeng, where the young shoots die, leading to the death of the entire top portion of the plant. In some cases, the infection progresses downward, reaching the stalk through the growing point. At advanced stages, the entire base of the shoot, including the growing point, becomes deformed. Leaves may show significant wrinkling, twisting, and rotting, with red specks and streaks appearing [24].

#### 2.2.3. Knife-Cut Stage

The knife-cut symptoms are typically seen in conjunction with the acute stage of the disease. They are marked by one or more horizontal cuts on the stem, resembling the effect of a sharp knife. This is a more extreme version of the characteristic ladder-like lesions seen in Pokkah Boeng disease [24].

**Table 1:** Pokkah Boeng disease causing *Fusarium* sp. in various hosts.

Pathogens	Morphology	Pigmentation	Host	Severity	Refs
*Fusarium proliferatum*	Soft and fluffy, macroconidia were hyaline, 3 to 5 septate, fusiform and micro conidia are ovoid, 0 to 1 septate, hyaline	Greyish-white to pale purple	Maize, sugarcane	High	[25,26]
*F. fujuikuroi*	Cottony growth, macroconidia were hyaline, 3 to 5 septate and micro conidia are ovoid, 0 to 1 septate	Purple	Maize, sugarcane	Extremely high	[27,28]
*F. verticilloides*	Cottony growth, macroconidia were hyaline, 3 to 5 septate and micro conidia are ovoid, 0 to 1 septate	Light pink to dark purple	Maize, sugarcane	Medium	[29]
*F. subglutinasis*	Cottony growth, macroconidia were hyaline, 3 to 5 septate and micro conidia are ovoid, 0 to 1 septate	Pinkish white or yellowish violet	Maize, sugarcane	Moderate	[30]
*F. sacchari*	Cottony growth, macroconidia were hyaline, 3 to 5 septate and micro conidia are ovoid, 0 to 1 septate	Pink to purple	Sugarcane	High	[31]
*F. andiyazi*	Cottony aerial mycelium, macroconidia were 3 to 5 septate and micro conidia are ovoid, 0 to 1 septate	Violet-colored	Sugarcane	Less	[32]

### 2.3. Environmental Factors Influence the Pokkah Boeng Disease

Pokkah Boeng disease is influenced by various environmental factors, plant management practices, and the quality of sugarcane setts. Stress factors such as water deficiency, temperature fluctuations, soil nutrient levels, and pH imbalances can all contribute to the susceptibility of sugarcane plants to the disease [33]. Hail damage weakens cane plants by bruising their stalks and breaking leaves, which makes them more susceptible to diseases as pathogens can easily enter through the damaged setts. Conditions that promote disease development include waterlogged soil, poor cultural practices leading to weed growth, continuous cultivation of the same variety in a field, and the presence of susceptible varieties in nearby areas [33].

Sandy clay loam soil with a pH range of 6.5–7.5 showed the highest occurrence of the disease compared to other soil types. It was also noted that the incidence of Pokkah Boeng increased as soil moisture levels rose. In terms of soil temperature, the study found that the disease thrived in temperatures between 24 and 29 °C, with the highest incidence occurring at 27.5 °C [34].

Temperature plays a key role in determining the spread of a pathogen, with optimal growth and sporulation occurring within the 20–30 °C range in both in vitro and in vivo conditions. The minimum, ideal, and maximum temperatures for pathogen growth are 10–15 °C, 30 °C, and 35–40 °C, respectively [22]. The disease was most severe when temperatures ranged from 20 °C to 32 °C, accompanied by high humidity levels of 70–80% and cloudy weather during the rainy season, particularly from July to September. Incidences of the disease were also observed in this period, with humidity levels between 79.0% and 85.5%, temperatures of 29.0–30 °C, and heavy rainfall. The ideal conditions for the growth of the *Fusarium* pathogen are temperatures between 20 and 30 °C and humidity levels of 75–85% [35].

The disease thrives in warm, moist conditions during the early monsoon, especially following summer showers and cloudy weather. Higher incidence of Pokkah Boeng disease was observed during the rainy season, from July to mid-September. In Bihar, favorable weather conditions for the disease’s growth included low temperatures, high humidity, and rainfall [36].

Nitrogen availability greatly influences the physiological and morphological traits of fungi, as well as the production of secondary metabolites such as fumonisin in *F. verticillioides* [37]. Fungi can adapt to both the quantity and type of nitrogen present through intricate regulatory systems [38]. Genome-wide microarray studies have shown that the expression of secondary metabolism (SM) genes in *G. fujikuroi* is affected by nitrogen availability, including the regulation of the polyketide synthase (PKS) gene cluster [39].

### 2.4. Life Cycle of Pokkah Boeng Disease Causing Fusarium sp.

**Figure 4:**
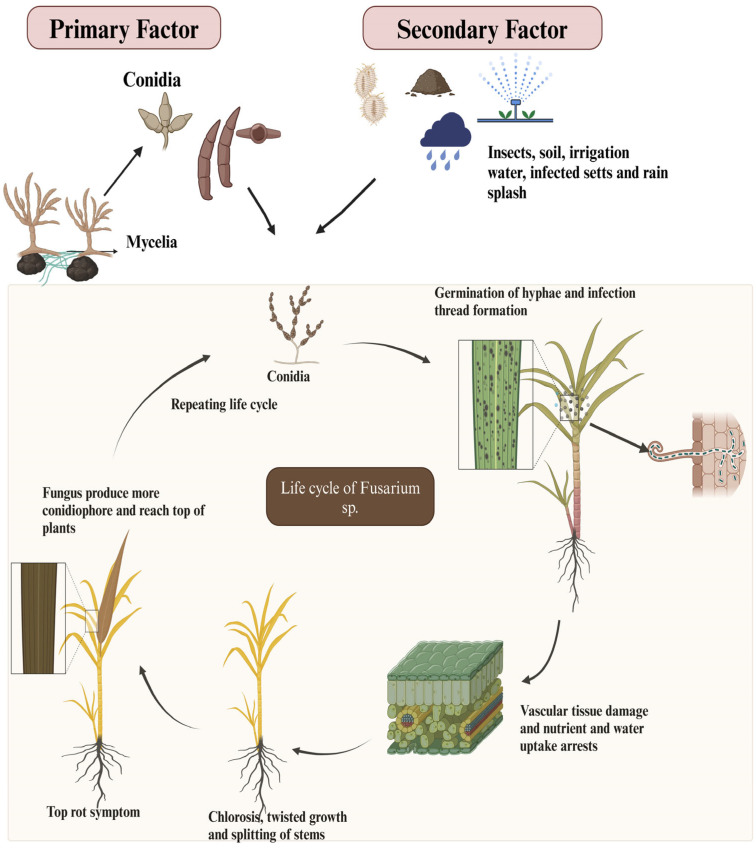
Life cycle of *Fusarium* in Pokkah Boeng disease.

Pokkah Boeng is an airborne disease spread through infected setts and rain splash. The secondary infection is spread through any injury by insects/borers or natural growth cracks. Conidia (asexual spores) (micro and macroconidia) are the primary means of reproduction and spread. Germination usually occurs on the surface of plant tissues. After the entry of the pathogen, the infection thread develops normal hypha which grows within the host tissues for some time. Once inside, the fungus grows in the vascular tissues, disrupting water and nutrient transport. This leads to characteristic symptoms such as chlorotic leaves, twisted growth, and splitting of the stalks. As the infection progresses, the fungus produces more conidiophores and reaches the top of the plant which causes the top rot symptom. Sexual reproduction may occur in some *Fusarium* species, leading to the formation of ascospores, although conidia are the primary means of spread [40]. The life cycle of *Fusarium*-causing Pokkah Boeng is explained in Figure 4.

The disease is primarily airborne; spreading through air circulation, and the pathogen, *F. moniliforme*, can survive for up to 12 months in plant debris under natural conditions and remain viable for over 10 months in laboratory settings. The fungus does not grow at 50 °C but can stay viable for at least six months. Studies have shown that *F. moniliforme* can survive for up to 12 months, though its incidence decreases after nine months. In natural conditions, the fungus survives for over 11 months at a soil depth of 30 cm. Cooler and drier conditions enhance its survival in plant debris [41].

The Pokkah Boeng pathogen spreads through the movement of spores carried by air currents, allowing it to colonize the leaves, flowers, and stems of plants. Overall, ratoon crops exhibited a higher incidence of mealybug infestation compared to plant crops. In some cases, mealybug infestation coincided with Pokkah Boeng disease, with 4 out of 17 fields (a 23.59% probability) experiencing a combined attack, intensifying the damage. Ratoon varieties Co 06022 and CoV 09356 showed particularly high levels of both mealybug infestation and Pokkah Boeng disease [42].

Both the pupae and adults of sugarcane stem borers can also spread the fungus [24]. The top borer, the Chilo species, often causes leaf distortion and shortening, symptoms that resemble those of Pokkah Boeng disease [43]. The secondary infections occur via infected setts, irrigation water, splashing rain, and soil.

## 3. Multitrophic Interaction of Pokkah Boeng, Mealybug, and Sugarcane

**Figure 5:**
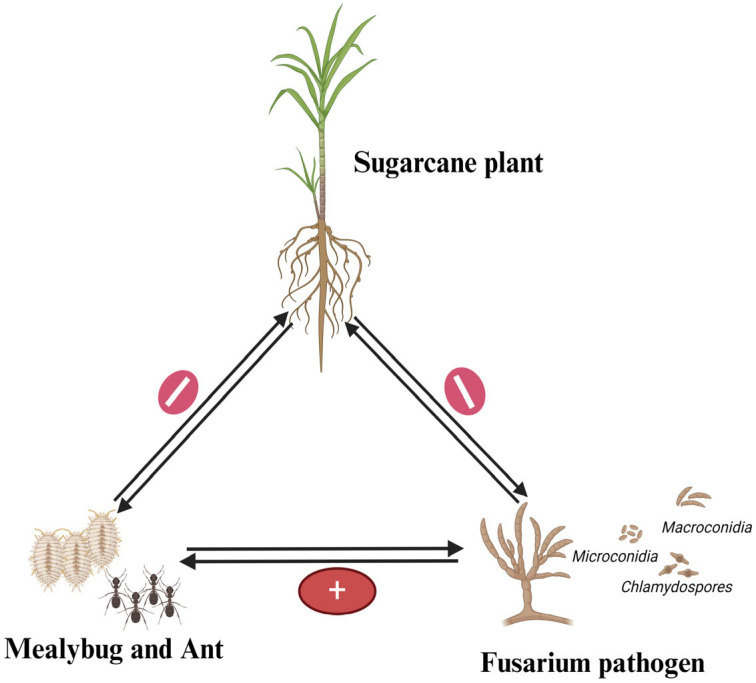
Tripartite interaction of Pokka Boeng, mealybug, and sugarcane.

One of the most extensively studied multitrophic interactions in sugarcane involves the complex relationship between the mealybug and Pokkah Boeng disease. This interaction occurs between *F. verticillioides* and the crown mealybug *Phenacoccus saccharifolii* (Green, 1908) (Phy.:Arthropoda, Class: Insecta, Fam.: Pseudococcidae). The presence of the mealybug in sugarcane fields is often linked with the pathogen, and their co-occurrence amplifies both the damage and spread of the disease in this patho system. The severity of Pokkah Boeng disease increases during the summer months (April to July) due to the presence of the crown mealybug *P. saccharifolii* in Tamil Nadu, India. The insect colonizes the crown region of the crop and is often associated with the black ant *Camponotus compressus* (Fabricius, 1787) (Phy.:Arthropoda, Class: Insecta, Fam.: Formicidae). The mealybug secretes large amounts of honeydew, which promotes the growth of sooty mold (*Capnodium* species) on the leaves (Figure 5). Crops aged three to seven months are most vulnerable to the disease, while in ratoon crops, even one-month-old plants can be affected by Pokkah Boeng [44].

## 4. Molecular-Based Detection of Pokkah Boeng Pathogen

Understanding the threshold pathogen density, variations in the pathogen’s distribution, and the interplay between the pathogen and the host are all necessary for developing effective disease control measures. Previously, pathogens in the infected samples were detected using polymerase chain reaction analysis owing to its low cost of the instrument and reagents. The detection of pathogens using real-time PCR has its own advantages, including high speed, improved sensitivity, and quantification of the pathogen load in the infected sample. The developed and evaluated PCR detection method used84 isolates of *Fusarium* and other fungal pathogens through PCR and real-time PCR [45]. The modified method had been used for detection of *Fusarium* strains [46], in which DNA was isolated from *Fusarium* strains followed by the sequencing of the ITS region and aligned with the already available datasets from NCBI.

Species from the *F. fujikuroi* species complex (FFSC) were identified in sugarcane plants showing symptoms of Pokkah Boeng in Brazil. This identification was based on two-loci molecular phylogeny, sexual compatibility, and the analysis of morphological markers. Among the 39 isolates studied, they identified *F. sacchari*, *F. proliferatum*, and an unknown genetic lineage closely related to *F. andiyazi*. When field isolates of *F. sacchari* and *F. proliferatum* were crossed with tester strains, they produced fertile perithecia and viable ascospores [31].

The RNAi silencing mechanism is a post-transcriptional gene silencing mechanism in which introduction of dsRNA causes gene silencing, and this technology emerged as a viable alternative to fungicides. Gene silencing of CYP51 and chitin synthase genes, viz., Chs1, Chs2, Chs3a, Chs3b, Chs4, Chs5, Chs6, and Chs7 results in reduction in infected spikelets [47]. Additionally, they reported reduction in mycotoxin levels produced by the pathogen in the transgenic lines.

## 5. Management of Pokkah Boeng Diseases

### 5.1. Cultural Methods

To manage Pokkah Boeng, it is recommended to plant resistant or moderately resistant varieties. Crop rotation should be practiced in fields affected by the disease to prevent its recurrence. Infected material, such as canes or setts showing symptoms of “top rot” or “knife cut”, should be promptly removed along with the entire clump, including the root system, and destroyed by burning. Early harvesting of diseased crops can help mitigate the spread. Additionally, ensure that only healthy setts or seed material are used for planting to reduce the risk of infection [48].

The variety CoG 6 suffered extreme damage due to both mealybug and Pokkah Boeng. Yuegan 49, Funong 11-2907, Mintang 11-610, Mintang 12-1404, and Guitang 11-1076 are resistant cultivars of China [49]. The Coimbatore variety that are resistant to Pokkah Boeng disease are Co 356, Co 976, Co 1053, Co 7204, Co 7717, Co 7910, Co 8210, CoJ 82315, Co 8318, Co 8339, Co 8341, Co 8353, CoS 86218, Co 87267, Co 87268, Co 87269, Co 87270, and Co 87271, from Cuddalore namely, C 79113, C 84028, and C 84070, from Andra Pradesh namely, CoA 88081 and CoA 89085, from Punjab namely, CoJ 46, CoJ 64, CoJ 72, CoJ 75, CoJ 76, CoJ 79, CoJ 82, CoJ 86, CoJ 89, CoJ 82191, CoJ 83536, CoJ 84191, and CoJ 99192 (CoJ 88), from Haryana namely, CoH 1, CoH 12, and CoH 13, and from Madhya Pradesh namely CoJn 80141, CoJn 86141, and CoJn 86310 [50].

The 0238 variety has been widely cultivated across 40% of the total farming area in Uttar Pradesh, gaining popularity due to its higher yield compared to other varieties. However, farmers recently discovered that its lower immunity makes it more susceptible to diseases such asPokkah Boeng. To combat this, a solution of two to three liters of buttermilk mixed with 100 L of water can be sprayed over the crops. This natural remedy helps control fungal growth and protects the plants [51].

Ratooning, also known as ‘stubble cropping’, refers to growing a new sugarcane crop from the regrowth of stubble left from the previous harvest without replanting new setts [52]. In India, it is typical to raise one to two ratoon crops, although cases of ‘multi-ratooning’ also exist. Over 50% of the sugarcane-growing area in the country is under ratoon cultivation [53], with the ratoon area being larger in subtropical regions compared to tropical ones.

### 5.2. Chemical Control

Fungicides offer both preventive and curative protection against various diseases. The testing of various fungicides to combat this disease remains an ongoing practice. In the early 1970s, pre-planting sett dips in Agallol and dithane Z-78 proved to be the most effective among seven fungicides tested.

Methyl benzimidazole carbamate (MBC) fungicides, particularly carbendazim, are broad-spectrum fungicides that effectively control fungal diseases across various crops. Carbendazim is particularly useful in preventing Pokkah Boeng disease, especially during the top rot phase of infection. In China, it has been widely used to manage Pokkah Boeng in chewing cane since the 1980s. However, continuous application of carbendazim in chewing cane fields is no longer recommended due to the emergence of resistant FSC (*Fusarium* species complex) [18].

The invention describes a method to prevent and treat sugarcane Pokkah Boeng disease. When no disease is present or early symptoms appear, a mixture of fluxapyroxad liquid, nano carbon liquid, and organosilicon is used. The fluxapyroxad concentration is 1.5–2.5 g/L, nano carbon concentration is 8–15 g/L, and organosilicon at 5–10 g/L are used to control Pokkah Boeng disease. The ideal application temperature is 20–26 °C, and the humidity should be 30–60%. This method improves chemical efficiency, with an overall prevention rate of more than 80%, making it highly effective and practical for widespread use [54].

The effectiveness of nine fungicides was evaluated, with carbendazim providing the highest disease control at 85.88%, followed by tebuconazole (83.18%), carbendazim + mancozeb (79.30%), propiconazole (77.11%), copper oxychloride (76.95%), hexaconazole (67.23%), mancozeb (61.42%), azoxystrobin (57.28%), and chlorothalonil (51.44%). A foliar spray of carbendazim resulted in the highest cane yield (92.68 t/ha), followed by tebuconazole (87.87 t/ha) [55].

The use of fungicides such as Bavistin, Blitox, copper oxychloride, and dithane M-45 for the management of Pokkah Boeng [22]. Similarly, carbendazim is used to control Pokkah Boeng disease [56]. Pokkah Boeng disease, caused by *F. moniliforme*, was effectively controlled by foliar application of fungicides such as 0.1–0.2% Bavistin and 0.1–0.2% Blitox-50, with four sprayings at intervals of 15 to 21 days. The disease caused an average loss in cane yield and commercial cane sugar of 15% to 25% [40].

Sett treatment with propiconazole 25% EC (1 mL/L) and imidacloprid 70 WS (1 mL/L) for 20 min as a preventive measure. Upon symptom appearance, spray a mixture of propiconazole 25% EC (1 mL), imidacloprid 17.8% SL (0.4 mL), a sticking agent (1 mL), and 1 L of water. If symptoms reappear, apply a spray of propiconazole 25% EC (1 mL/L) combined with either flonicamid 50 WG (0.3 g/L) or clothianidine 50 WDG (0.5 g/L) at 20-day intervals [36]. The chemicals fungicides used for the control of Pokkah Boeng disease are depicted in Table 2.

### 5.3. Biological Control Agents

The growing consumer demand for pesticide-free food, coupled with the increasing cost of pesticides, has driven the search for alternative solutions. As a result, there is a pressing need to develop affordable and environmentally friendly non-chemical methods for managing plant diseases. This led to the replacement of synthetic fungicides with biological control strategies, where the use of antagonistic microorganisms to combat diseases has proven highly effective [59,60].

Currently, biological control of plant diseases primarily depends on beneficial microorganisms and their metabolites. These beneficial microorganisms—bacteria, actinobacteria, fungi, and viruses play a crucial role in maintaining plant health and safety [61,62].

**Figure 6:**
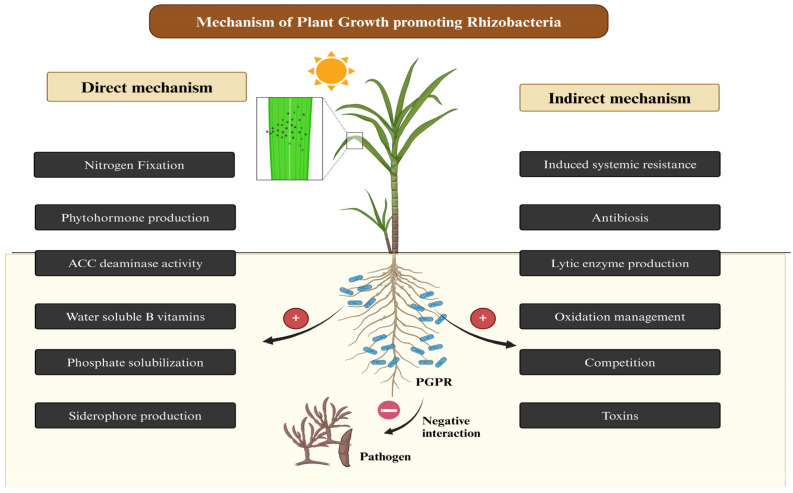
Mechanism of plant growth promoting rhizobacteria for disease control.

Microbial biocontrol agents employ a variety of strategies to protect plants from pathogen infections. They can use one or multiple mechanisms to prevent or mitigate plant diseases, either by directly or indirectly interacting with the pathogen. BCAs can directly engage with pathogens by secreting antimicrobial compounds, disrupting pathogen virulence, and competing for nutrients and space (Figure 6). Many microbes produce and release metabolites such as lipopeptides, bacteriocins, antibiotics, biosurfactants, cell-wall-degrading enzymes, or microbial volatile compounds, all of which possess antimicrobial properties that inhibit the growth or metabolic activity of pathogens [63].

The crude biosurfactant rhamnolipid from *Pseudomonas aeruginosa* DS9 effectively inhibited the growth of the plant pathogen *F. sacchari*, with the highest concentration (2.0 g/L) significantly reducing fungal growth by more than 50%, showing a stronger effect than the cell-free culture supernatant or whole culture treatment [64]. Additionally, several bacteria isolated from sugarcane (*Nguyenibacter vanlangensis*, *Acidomonas methanolica*, *Asaia bogorensis*, *Tanticharoenia aidae*, *Burkholderia gladioli*, and *Bacillus altitudinis*) exhibited strong antagonistic activity against *F. moniliforme*. These bacteria not only controlled diseases, but also promoted plant growth by producing growth-promoting substances such as IAA, siderophores, ammonia, and by solubilizing zinc or phosphate [65]. Studies on endophytic microbes in sugarcane varieties with high resistance (HR) and high susceptibility (HS) revealed that HR varieties had a higher abundance of beneficial fungi, such as *Shinella*, *Dechloromonas* and *Microbacter*, and the lowest population of harmful fungi, such as *Fusarium*, *Ramichloridium*, and *Scleroramularia* [66]. A combination of phosphite (Phi) and *Trichoderma* (Taz-016) effectively controlled *Fusarium* in sugarcane, with 4000 µg/mL Phi and *Trichoderma* Taz-016, resulting in improved plant height, stem length, and leaf dry weight [67]. Field studies also showed that sett treatments with *Pseudomonas fluorescens* (Psf02) achieved the lowest disease incidence at 0.92%, followed by the consortium of *Trichoderma* Th14 and *P. fluorescens* Psf02 at 1.51%, and Th14 alone at 5.53% [68]. The biological potential of microbial agents and their mode of action are explained in Table 3.

## 6. Conclusions

Pokkah Boeng disease in sugarcane continues to pose significant challenges for sustainable sugarcane production, affecting both yield and quality. This review of the disease highlights the complex interaction between environmental factors, pathogen behavior, and plant susceptibility. Current management practices, including the use of resistant varieties, timely fungicide applications, and integrated pest management (IPM) strategies, have shown promise but require further refinement to ensure long-term efficacy. Looking ahead, there is a need for more in-depth research into the genetic resistance mechanisms in sugarcane, alongside the development of new, targeted fungicides that are environmentally friendly. Advances in biotechnology, such as gene editing and molecular breeding, hold potential for creating more resilient sugarcane varieties. Moreover, improving early detection systems and disease forecasting models will enable farmers to take timely preventive measures, minimizing the economic impact of Pokkah Boeng. Effective management of Pokkah Boeng disease will require a combination of traditional and innovative approaches, emphasizing collaboration between researchers, industry stakeholders, and farmers. By integrating new scientific discoveries with practical agricultural practices, the sugarcane industry can move towards sustainable disease management, securing the future of this vital crop.

## Figures and Tables

**Table 2:** Fungicides effective against PokkahBoeng disease in sugarcane.

Common Name	Trade Name	Chemical Group/Class	References
Carbendazim	Bavistin	Benzimidazole	[18]
Copper oxychloride	Blitox	Inorganic chemical compound	[56]
Mancozeb	Dithane	Ethylenebisdithiocarbamate	[55]
Propiconazole	Zerox	Triazole fungicide	[55]
Tebuconazole	Folicur	Triazole fungicide	[57]
Hexaconazole	Alert Creeper	Triazole fungicide	[55]
Azoxystrobulin	Amistar	Methoxyacrylate class of organic compounds	[58]
Chlorothalonil	Bravo	Organochlorine fungicide	[55]

**Table 3:** Biocontrol potential of microbial control agents against pathogens.

Biocontrol Agent	*Fusarium* Pathogens	Mode of Action	References
*B. subtilis*	*F. verticillioides*	Produce chitinase and glucanase	[69]
*Burkholderia* sp.	*F. verticillioides*, *F. proliferatum*	NR	[70]
*P. fluorescens*	*F. verticillioides*	Antifungal activity and induced systemic resistance (ISR)	[71]
*T. viride* and *T. harzianum*	*F. fujikuroi* and *F. proliferatum*	Competition for food and space	[72]
*Paenibacillus polymyxa*	*F. verticillioides*	NR	[73]
*P. aeruginosa*	*F.sacchari*	Antifungal activity	[64]
*T. harzianum*	*Fusarium* sp.	Hyperparasitism	[50]
*B. amyloliquefaciens*	*F. sacchari*	NR	[33]

NR—Not reported.
